# Are symptoms assessed differently for schizophrenia and other psychoses in legal insanity evaluations of violent crimes?

**DOI:** 10.1186/s12888-023-04992-6

**Published:** 2023-07-07

**Authors:** Pia Jorde Løvgren, Petter Laake, Kjersti Narud, Solveig Klæbo Reitan, Stål Bjørkly

**Affiliations:** 1 Faculty of Medicine, University of Oslo, Oslo, Norway; 2 The Regional Centre for Research and Education in Forensic Psychiatry for the South-Eastern Norway Regional Health Authority, Oslo University Hospital, Oslo, Norway; 3 Department of Biostatistics, Oslo Centre for Biostatistics and Epidemiology, University of Oslo, Oslo, Norway; 4 Department of mental health, Faculty of Medicine and Health Science, Norwegian University of Science and Technology, Trondheim, Norway; 5 Nidelv Center of Community Mental Health, St. Olav’s Hospital, Molde, Norway; 6 Faculty of Health and Social Sciences, Molde University College, Molde, Norway

**Keywords:** Delusions, Hallucinations, Forensic, Insanity, Bias, Stigma

## Abstract

**Background:**

Forensic evaluations of legal insanity include the experts’ assessment of symptoms present at the mental state examination (MSE) and the mental state at the time of offense (MSO). Delusions and hallucinations are most important. We explored how often symptoms were recorded in written forensic reports.

**Design:**

This exploratory, cross-sectional study included 500 reports of legal insanity written in 2009–2018 from cases of violent crimes in Norway. The first author read all reports and coded symptoms recorded from the experts’ assessments of the offenders. Two co-authors repeated this procedure for 50 randomly selected reports. Interrater reliability was calculated with Gwet’s AC_1_. Generalized Linear Mixed Models with Wald tests for fixed effects and Risk Ratios as effect sizes were used for the statistical analyses.

**Results:**

Legal insanity was the main conclusion in 23.6% of the reports; 71.2% of these were diagnosed with schizophrenia while 22.9% had other psychotic disorders. Experts recorded few symptoms from MSO, but more from MSE, although MSO is important for insanity. We found a significant association between delusions and hallucinations recorded present in the MSO and legal insanity for defendants with other psychotic disorders, but no association for defendants with schizophrenia. The differences in symptom recordings between diagnoses were significant.

**Conclusion:**

Few symptoms were recorded from the MSO. We found no association between presence of delusions or hallucinations and legal insanity for defendants with schizophrenia. This may indicate that a schizophrenia diagnosis is more important to the forensic conclusion than the symptoms recorded in the MSO.

## Introduction

The concept of legal insanity, i.e., that certain mental conditions with severe impairment should exculpate one of moral and legal guilt, dates back to our earliest societies [1–4]. A prerequisite for legal insanity is the presence of a severe mental disorder that grossly impairs a defendant’s perception and understanding of reality at the time of the alleged offense [1, 3, 5–9].

In Norway, to be criminally responsible, you have to be over 15 years, and not have any severe mental disorders at the time of the crime. The mental disorder part of criminal responsibility is three-pronged: An offender could be judged not responsible if he or she was psychotic (as a legal, not a medical term)^1^; if he or she had a mental retardation; or if he or she had a severe disturbance of consciousness at the time of the crime [10]. The first prong refers to conditions where the defendant has a severely reduced capacity to understand his or her relationship with their surroundings, which most often equal psychotic conditions. This prong is like the term “legal insanity”. As this expression is well known, we use this latter expression in this paper, as the term “psychotic” could easily be confused with the medical term. The second prong refers to mental defects that affect the defendant´s understanding of his or her actions with respect to the law, i.e. an intellectual deficit. The third resembles the term “automatism”, which is more known internationally, and refers to a state where a person is not in full control of his or her actions because a reduced conscious awareness caused by physiological changes distorts the perception and control of actions [11].

Norway has a forensic system that differs from most other systems in that the legal rule of criminal responsibility is based on a medical or biological principle; a defendant could be excused if he or she had a legally relevant mental condition at the time of the crime regardless of the relationship between the condition and the criminal act [10]. At the time when the reports included in our sample were written, forensic experts were asked to give an ultimate opinion on whether an offender was criminally irresponsible^2^ [12].

Mental health professionals play an important role in informing the judicial system which persons and conditions should be exempted from criminal liability by conducting forensic psychiatric evaluations [4, 13]. Information from these evaluations is presented in written reports, in which the professionals must provide clear, concise evidence to assist the courts in ruling in questions of legal insanity [1, 14, 15]. The reports include assessments of the defendant’s mental state at the time of the examination (MSE) and at the time of the offense (MSO).

Though legal regulations differ between countries, the ground principle of the existence of a severe mental disorder is always a prerequisite for legal insanity. This means that forensic evaluations in these cases must establish whether a mental disorder or defect was present at the time of the crime, and whether this condition affected the offender’s capacity for judgment when the crime was committed [3, 5, 10, 16]. To achieve this, the experts should record the defendant’s emotions, cognition, and behavior right before, during, and after the time of the offense. As most legally insane defendants have a psychosis, the descriptions of symptoms of psychosis are essential to this evaluation. However, since the most important time point for the evaluation is the time of the crime,^3^ these evaluations are retrospective, which makes them challenging to perform [3, 7, 17].

The usefulness of diagnoses in forensic evaluations of criminal responsibility has been debated [1, 3, 8]. The most common view is that diagnoses could be helpful in understanding the degree of impairment and could give a framework for collecting data regarding MSO [3]. The great majority of studies have found that a psychotic disorder is often required for courts to give a ruling of legal insanity [3, 18–20], with schizophrenia being the most frequent diagnostic category [19, 21–28]. However, almost all sources emphasize that it is not the diagnostic category but the mental condition, symptoms and functional impairment that is important to the insanity evaluation [1, 3, 9, 10]. Thus, experts should record which symptoms were present or not at the time of the crime.

The literature indicates that forensic experts are prone to bias and errors in their evaluations [1, 29–31]. A common error is for experts to give the diagnosis too much emphasis at the expense of describing the defendant’s symptoms and level of functioning [16, 24, 32–34]. Some have claimed that experts have difficulty distinguishing between MSE and MSO in their evaluations, and that they focus on symptoms that are present at the time of the examination more than is justified to make inferences regarding MSO [1, 18, 35]. Others emphasize that experts do not always explain how the data they collect relate to their forensic opinions [1, 36], while still others point out that experts either do not collect enough data or do not record these data in their reports [1, 37].

When treatment options for schizophrenia were less effective, the illness often had a severe course. Individuals with schizophrenia were believed to be unable to take responsibility for their actions in all aspects of life [25]. Contemporary views are that the course of schizophrenia differs both between individuals and within each individual, with episodes of exacerbation that intercede with periods of remission; thus, schizophrenia is no longer automatically associated with insanity [24, 26, 32]. Indeed, it is necessary to demonstrate that the defendant´s mental condition was in an active phase with severe symptoms that disrupted behavior at the time of the crime in order to be considered legally insane [1, 3, 8, 10, 26, 38]. Delusions and hallucinations have the greatest potential to destabilize, as they generate distorted beliefs and perceptions of reality, and provide irrational reasons for actions [9, 18, 19, 37–39]. Thus, these symptoms are among the most important ones to describe.

Although there is widespread acceptance that symptoms are more important than diagnoses regarding legal insanity, few studies have focused on how defendants’ symptoms are described in forensic reports. In one study, 75% of legally insane defendants were diagnosed with schizophrenia. In almost 90% of these defendants, delusions were recorded as present. About 50% suffered from command hallucinations, and nearly 25% had formal thought disorder at the time of the offense [23]. However, as the study included 40 subjects, the results may be uncertain due to low sample size. Another study reported that although delusions were the most common factor linking mental illness to the offense, only 64% of reports recorded delusions as present at the time of the crime [21]. Other studies have also suggested that clinical descriptions were lacking in reports of forensic evaluations, although they focused on the overall quality of reports, not on the presence of symptoms [14, 40].

The overarching aim of our study is to explore factors that might be related to higher quality in written reports of forensic evaluations of legal insanity. We wanted to investigate the assumption mentioned in the literature, that a common error in forensic evaluations is to put more emphasis on diagnosis at the expense of referring to the symptoms present at the time of the crime. Thus, we explored the experts’ recordings of symptoms of psychosis in forensic reports, investigated the link between different psychosis diagnoses and legal insanity, and analyzed whether we could find evidence suggesting which was more important for the insanity evaluation; symptoms or diagnoses. We investigated:

1. Diagnostic categories in legal insanity evaluations and their associations with opinions of legal insanity.
2. The recordings of symptoms of psychosis in the reports.
3. The correlations between symptoms of psychosis recorded as present at the time of the offense and the opinion of legal insanity for schizophrenia versus other psychosis diagnoses.

## Methods

### Study design

This exploratory, cross-sectional study of registry data was based on 500 anonymized reports of forensic evaluations of criminal responsibility in court cases of severe violent offenders, and were collected from the archives of the Norwegian Board of Forensic Medicine (NBFM). We selected 100 reports per year for the years 2009, 2011, 2013, 2015, and 2018, to reach a sufficient sample size per year and cover a longer period [41]. The selection criterion for the reports was the index offense, which the NBFM uses as one of the sorting systems in their archives. Thus, we searched for the penal codes in the register of reports. We selected reports written in the cases with the most serious violent crimes first, but added less severe crimes to obtain a number of 100 per year. These were murder in 79 reports (15.8%), attempted murder in 72 reports (14.4%), violence or severe violent threats in 279 reports (55.8%), sexual crimes in 63 reports (12.6%), and other crimes in 7 reports (1.4%).

### Data acquisition

We designed a registration form for this study and collected data from May 2019 until May 2022. The first author (PJL) read all reports and extracted symptom information from the experts’ assessments of MSE and MSO. Two co-authors (KN and SKR) repeated this procedure for 50 randomly selected reports to determine inter-rater reliability.

In Norway, teams of either two psychiatrists, or one psychiatrist and one psychologist, are appointed by the court to conduct evaluations of criminal responsibility. It is mandatory for the experts to send the report to the Norwegian Board of Forensic Medicine for formal control, and the experts must submit additional reports if there are parts that are missing or inadequately investigated.

All reports are structured in mostly the same way. They start with a part where the mandate is presented together with information regarding the offense and collateral information from police records. Second comes a part with recordings of the expert authors’ conversations with the defendant, including any testing or structured interviews, and a clinical evaluation. This information is the basis for the experts’ mental state examination of the defendant and provided the information regarding MSE in our study. Thirdly, any other collateral information, such as medical records, interviews with relatives and others, is referred. The last part is where the expert authors describe their diagnostic and legal considerations regarding the defendant’s mental state at the time of the offense. This part provided the information regarding MSO in our study. The last page is a summary of the conclusions. The experts collect information from all collateral sources, as well as from the interviews with the defendant to obtain information to assess the MSO in the defendant.

In our study, we collected symptom information from recordings of interviews between experts and defendants, experts’ examination of MSE, and experts’ written discussion of the diagnostic conclusions (recorded using the International Classification of Diseases, Revision 10) [42] and forensic conclusions. These parts constituted 20–50% of the reports’ pages. During the study period, Norwegian experts were to state whether they considered the defendant to have an active psychotic state at the time of the examination, and to give their opinion as to whether the defendant was criminally responsible at the time of the offense. The final ruling on the question of responsibility is done by the court; however, we did not collect information regarding the final rulings.

Some teams of experts wrote many reports, while others wrote few. This led to a statistical dependence between the observations collected from reports written by the same team. Thus, we had to design analyses that controlled for this nesting in our sample.

### Symptom assessment

The Positive and Negative Syndrome Scale (PANSS) [43] is one of the most well-known and widely used instruments for assessing symptoms in schizophrenia. It was designed for research but is also used in clinical settings [44]. However, it was not designed for assessing symptoms in written records. We performed a pilot study to evaluate the method before commencing the main study. In the pilot study, three experienced forensic psychiatrists (main author PJL and co-authors SKR and KN) assessed PANSS items in 20 reports for interrater reliability measures. We achieved high interrater reliability, with a mean value of Gwet’s AC_1_ of 0.805 [45], which is considered a very high agreement [46].

For the main study, we used the seven positive items in the PANSS to collect symptom information. In order to analyze symptoms associated with forensic conclusions, we chose items that are particularly relevant to the assessment of legal insanity: P1 delusions, P2 conceptual disorganization (thought disorder), and P3 hallucinatory behavior.

We developed a codebook for the assessment based on the PANSS manual and on the results of the pilot study. In the pilot study we discovered that experts often used the collective terms “psychotic symptoms” and “impaired level of function” to describe defendants’ mental states. These terms were written words and constructs, and the content of these was not further defined. Nevertheless, as they were used often, we decided to include them in our analyses to get a better picture of the experts' symptom descriptions than could be obtained through the PANSS items alone. Thus, we revised the codebook and added these collective terms.

The PANSS items were not categorized using the PANSS rating scale. Instead, we categorized PANSS items and collective terms as “recorded as present”, “recorded as not present”, or “not mentioned” in experts’ assessment of MSE and MSO.

### Statistics

Descriptive statistics are presented as counts and percentages. Associations between symptom descriptions and forensic conclusions were analyzed in stratified, two-way contingency tables. Risk ratio (RR) was used as an effect measure. Due to statistical dependence between the reports written by the same teams, risk ratios with 95% confidence intervals (CI) and overall p-values were estimated in generalized linear mixed models (GLMMs), with log links. All test statistics were Wald tests.

Post hoc statistical power analyses were not performed. Given observed effect sizes, p-values, and confidence intervals, post hoc power analyses provide no additional information [47].

Interrater reliability measures were analyzed using Gwet’s AC_1_ [45, 48, 49]. Data were analyzed with IBM SPSS Statistics tools version 25, and the GLMM in STATA16. We used a significance level of 5%.

### Ethics

An ethical statement is provided in the Declarations section.

## Results

### Descriptive statistics regarding reports

The 500 reports were written by 243 pairs of experts. Of these pairs, 65% wrote one report and 82% wrote two or fewer reports. The maximum number of reports written by one pair was 29. The number of total reports written in Norway increased over this period. Our sample of 100 reports accounted for 32.2% of the total sample of reports written in 2009, and for 20.9% in 2018.

The reports varied in length. The range was from 6 to 151 pages (mean: 32.55, SD: 13.75; median: 30.00, interquartile range: 24.00–38.75).

All reports included collateral information from police records regarding the index offence, while 72.2% included information from hospitals, 31.0% from general practitioners, and 33.0% included interviews with collateral sources, like spouses, family and others.

The defendant cooperated with the experts in 90.8% of the reports. The time interval from the crime committed to the first interview the experts conducted with the defendants varied. The range was from 0 to 1024 weeks (mean: 33.68; SD: 79.79; median: 16.00; interquartile range 8.00–35.00).

### Aim 1: diagnostic categories in evaluations of legal insanity

A diagnosis in the psychotic spectrum was given in 191 reports (38.2%). Schizophrenia was the principal diagnosis in 100 reports (20.0%), other psychotic disorders in 49 reports (9.8%), and substance-induced psychotic disorders (SIPD) in 42 reports (8.4%) (Table 1).

**Table 1:** Prevalence of selected diagnostic categories in reports of forensic evaluations of criminal responsibility of cases regarding violent crime, *n* = 500

Diagnostic categories		Legal insanity		Not responsible for other causes	No conclusion	Total
		Yes	No			
Schizophrenia	n (%)	84 (84.0)	16 (16.0)	0 (0)	0 (0)	100 (100)
*F20.0 Paranoid*	n (%)	72 (85.7)	12 (14.3)	0 (0)	0 (0)	84 (100)
* F20.1 Hebephrenic*	n (%)	5 (71.4)	2 (28.6)	0 (0)	0 (0)	7 (100)
* F20.3 Undifferentiated*	n (%)	5 (71.4)	2 (28.6)	0 (0)	0 (0)	7 (100)
* F20.9 Unspecified*	n (%)	2 (100)	0 (0)	0 (0)	0 (0)	2 (100)
Other psychotic disorders	n (%)	27 (55.1)	22 (44.9)	0 (0)	0 (0)	49 (100)
* F21 Schizotypal disorders*	n (%)	1 (20.0)	4 (80.0)	0 (0)	0 (0)	5 (100)
* F22.0 Paranoid psychosis*	n (%)	14 (66.7)	7 (33.3)	0 (0)	0 (0)	21 (100)
* F2- Other psychoses*^a^	n (%)	1 (12.5)	7 (87.5)	0 (0)	0 (0)	8 (100)
* F3- Affective psychosis*^b^	n (%)	11 (73.3)	4 (26.7)	0 (0)	0 (0)	15 (100)
SIPD^c^	n (%)	0 (0)	41 (97.6)	1^e^ (2.4)	0 (0)	42 (100)
Other diagnoses^d^	n (%)	6^f^ (2.4)	231 (92.4)	9^ g^ (3.6)	4^ h^ (1.6)	250 (100)
No diagnosis	n (%)	1^i^ (1.7)	48 (81.4)	1 (1.7)	9 (15.2)	59 (100)
Total		118 (23.6)	358 (71.6)	11 (2.2)	13 (2.6)	500 (100)

^a^F23.2 (1), F23.3 (1), F28 (1), F29 (5). ^b^F25 (10), F31.2 (4), F32.3 (1). ^c^Substance-induced psychotic disorder, F10.5 (1), F12.5 (4), F15.5 (5), F19.5 (31), F19.7 (1). ^d^All other diagnostic categories than psychosis in the International Classification of Diseases, Revision 10. ^e^Strong disturbance of consciousness (automatism): F19.5. ^f^F06.7, F11.25, F15.6, F31.6, F32.2, F84.9. ^g^Severe mental retardation: F70, F71. ^h^F19.21, F60.2, F60.9. ^i^Concluding with a probable schizophrenia diagnosis, but not confirmed

An opinion of criminal irresponsibility was given in 25.8% of the reports. Legal insanity (psychotic) was the conclusion in 23.6%, severe mental retardation in 2.0%, and severe disturbance of consciousness in 0.2%. Experts considered 84 (84%) of defendants diagnosed with schizophrenia and 27 (55.1%) of defendants with other psychotic disorders, to be legally insane, respectively, while none of defendants diagnosed with SIPD were considered insane. Among all defendants considered legally insane by the experts, 71.2% had schizophrenia and 22.9% had any other psychotic disorder.

The likelihood of being considered legally insane was 52% higher for defendants with a schizophrenia diagnosis than for defendants diagnosed with any other psychotic disorder (RR = 1.52, 95% CI: 1.18–1.97, *p* = 0.001). The likelihood of being considered legally insane was 29% higher when a defendant was diagnosed with paranoid schizophrenia than with paranoid psychosis (RR = 1.29, 95% CI: 0.93–1.78, *p* = 0.131), which is not statistically significant (results not shown in table).

### Aim 2: symptoms of psychosis recorded in evaluations of legal insanity

The presence or absence of symptoms was more often recorded in the assessment of MSE than of MSO. The symptoms most often recorded in assessments of MSE were hallucinatory behavior (in 87.6% of the reports), followed by delusions (84.4%), and the collective term “psychotic symptoms” (81.0%). The symptoms most often recorded in assessments of MSO were “psychotic symptoms” (66.6%), followed by delusions (28.0%), and hallucinatory behavior (23.0%) (Table 2).

**Table 2:** Symptoms mentioned in the assessments of mental state at the time of the examination and at the time of the offense in cases of violent crime, *n* = 500

	Recorded as present		Recorded as absent		Not mentioned	
	Count	%	Count	%	Count	%
Symptoms at the time of the examination						
P1 Delusions^a^	106	(21.2)	316	(63.2)	78	(15.6)
P2 Conceptual disorganization	73	(14.6)	317	(63.4)	110	(22.0)
P3 Hallucinatory behavior	67	(13.4)	371	(74.2)	62	(12.4)
P4 Excitement	36	(7.2)	150	(30.0)	314	(62.8)
P5 Grandiosity	44	(8.8)	62	(12.4)	394	(78.8)
P6 Suspiciousness	95	(19.0)	102	(20.4)	303	(60.6)
P7 Hostility	11	(2.2)	140	(28.0)	349	(69.8)
Psychotic symptoms^b^	90	(18.0)	315	(63.0)	95	(19.0)
Impaired level of function^b^	122	(24.4)	16	(3.2)	362	(72.4)
Symptoms at the time of the offense						
P1 Delusions	84	(16.8)	56	(11.2)	360	(72.0)
P2 Conceptual disorganization	30	(6.0)	43	(8.6)	427	(85.4)
P3 Hallucinatory behavior	48	(9.6)	67	(13.4)	385	(77.0)
P4 Excitement	44	(8.8)	4	(0.8)	452	(90.4)
P5 Grandiosity	9	(1.8)	1	(0.2)	490	(98.0)
P6 Suspiciousness	39	(7.8)	2	(0.4)	459	(91.8)
P7 Hostility	4	(0.8)	-	-	496	(99.2)
Psychotic symptoms^b^	109	(21.8)	224	(44.8)	167	(33.4)
Impaired level of function^b^	102	(20.4)	12	(2.4)	386	(77.2)

^a^P1-P7 are the positive items from the instrument PANSS (se article text for reference). ^b^Collective terms for symptom descriptions often used by experts in their written reports

We found no significant changes over time in how often delusions, conceptual disorganization, or hallucinatory behavior were mentioned. However, mentioning of the term “psychotic symptoms” showed significant variation in the assessment of MSE over time (chi-square = 10.6, df = 4, *p* = 0.032), with significantly more frequent mentioning in 2018 compared with 2009 (RR = 2.05, 95% CI: 1.30–3.22, *p* = 0.002). There was no significant variation in the mentioning of “psychotic symptoms” over time in assessment of MSO (chi-square = 4.5, df = 4, *p* = 0.34) (not shown in table).

### Aim 3: associations between the presence or absence of symptoms in assessments of MSE, MSO, and the opinion of legal insanity by diagnostic category

When delusions were recorded as present in the assessment of MSE, 88.2% of defendants with schizophrenia, and 61.3% of defendants with other psychotic disorders were reported to have an active psychotic state. When delusions were recorded as absent or were not mentioned at all in the assessment of MSE, 43.8% of defendants with schizophrenia and 3.3% of defendants with other psychotic disorders were reported to have an active psychotic state (Table 3). Thus, the probability that a defendant with schizophrenia was reported to have an active psychotic state at the time of examination was two times higher if delusions were present in the assessment of MSE (RR = 2.02, 95% CI: 1.35–3.01, *p* = 0.001). For a defendant diagnosed with any other psychotic disorder, the probability of being reported to have an active psychotic state at the time of examination was 18.4 times higher if delusions were present (RR = 18.4, 95% CI: 4.40–76.7 *p* < 0.001). The risk ratios were significantly higher for other psychotic disorders than for schizophrenia for all symptoms recorded as present in the assessment of MSE, except for the construct “impaired level of function”.

**Table 3:** Associations between symptoms recorded in the assessment of mental state at the time of examination and active psychotic state in cases of violent crime, stratified by schizophrenia and other psychotic disorders, n = 191, risk ratios (RR) estimated by generalized linear mixed model

		Time of examination					
		Schizophrenia			Other psychotic disorders^c^		
Symptoms		Active psychotic state^a^		RR^b^ 95%CI	Active psychotic state		RR 95% CI
		Yes	No		Yes	No	
P1 Delusions	Present	60 (88.2)	8 (11.8)	2.02^**^	19 (61.3)	12 (38.7)	18.4^***^
	Not/no info^d^	14 (43.8)	18 (56.3)	[1.35–3.01]	2 (3.3)	58 (96.7)	[4.40–76.7]
P2 Conceptual disorganization	Present	49 (87.5)	7 (12.5)	1.54^**^	9 (64.3)	5 (35.7)	4.13^***^
	Not/no info	25 (56.8)	19 (43.2)	[1.19–1.99]	12 (15.6)	65 (84.4)	[2.19–7.77]
P3 Hallucina-tory behavior	Present	47 (90.4)	5 (9.6)	1.61^***^	8 (80.0)	2 (20.0)	4.98^***^
	Not/no info	27 (55.2)	21 (43.8)	[1.25–2.07]	13 (16.0)	68 (84.0)	[2.87–8.65]
Psychotic symptoms^e^	Present	55 (88.7)	7 (11.3)	1.77^**^	14 (66.7)	7 (33.3)	6.67^***^
	Not/no info	19 (50.0)	19 (50.0)	[1.28–2.46]	7 (10.0)	63 (90.0)	[2.81–15.8]
Impaired level of function^e^	Present	44 (84.6)	8 (15.4)	1.35^*^	7 (29.2)	17 (70.8)	1.40
	Not/no info	30 (62.5)	18 (37.5)	[1.05–1.75]	14 (20.9)	53 (79.1)	[0.61–3.20]

^a^This refers to the conclusion of whether the defendant had an active psychotic state of illness (equivalent to legal insanity) at the time of the mental examination (MSE). ^b^RR = Risk ratio. ^c^All other diagnoses from F2 and F3-chapters, including SIPD, see Table 1. ^d^This category includes symptoms recorded as absent, and symptoms not mentioned. ^e^These are general terms used by the forensic experts in their description of the defendants’ mental state

^*^p < 0.05, **p < 0.01, ***p < 0.001

When delusions were recorded as present in the assessment of MSO, experts gave an opinion of legal insanity for 89.2% of defendants with schizophrenia and 60.5% of defendants with other psychotic disorders. When delusions were recorded as absent or not mentioned at all in the MSO, experts gave an opinion of legal insanity for 81.0% of defendants with schizophrenia and 9.4% of defendants with other psychotic disorders (Table 4). There was no higher probability that a defendant with schizophrenia would be considered legally insane when delusions were recorded as present in the MSO compared to when delusions were recorded as absent or not mentioned at all. For defendants with other psychotic disorders, the probability of being considered legally insane was 6.42 times higher if delusions were recorded as present in the MSO (RR = 6.42, 95% CI: 2.64–15.6, *p* < 0.001). We found significantly higher risk ratios for defendants with other psychotic disorders compared to defendants with schizophrenia for delusions, conceptual disorganization, and impaired level of function: Delusions (RR 5.82; 95% CI: 2.41–14.05, *p* < 0.001), conceptual disorganization (RR 2.08; 95% CI: 1.08–4.00, *p* < 0.001), “impaired level of function” (RR 2.13; 95% CI: 1.04–4.36, *p* = 0.039).

**Table 4:** Association between symptoms recorded in the assessment of mental state at the time of the offense and legal insanity in cases of violent crime, stratified by schizophrenia and other psychotic disorders, *n* = 191, risk ratios (RR) estimated by generalized linear mixed model

		Time of offense					
		Schizophrenia			Other psychotic disorders^c^		
Symptoms		Legal insanity^a^		RR^b^ 95%CI	Legal insanity		RR 95% CI
		Yes	No		Yes	No	
P1 Delusions	Present	33 (89.2)	4 (10.8)	1.10	23 (60.5)	15 (39.5)	6.42***
	Not/no info^d^	51 (81.0)	12 (19.0)	[0.94–1.30]	5 (9.4)	48 (90.6)	[2.64–15.6]
P2 Conceptual disorganization	Present	15 (88.2)	2 (11.8)	1.06	6 (60.0)	4 (40.0)	2.21*
	Not/no info	69 (83.1)	14 (16.9)	[0.87–1.29]	22 (27.2)	59 (72.8)	[1.17–4.17]
P3 Hallucina-tory behavior	Present	26 (92.9)	2 (7.1)	1.15	8 (53.3)	7 (46.7)	2.03*
	Not/no info	58 (80.6)	14 (19.4)	[0.99–1.35]	20 (26.3)	56 (73.7)	[1.16–3.53]
Psychotic symptoms^e^	Present	61 (92.4)	5 (7.6)	1.37*	15 (48.4)	16 (21.6)	2.23**
	Not/no info	23 (67.6)	11 (32.4)	[1.05–1.78]	13 (21.7)	47 (78.3)	[1.22–4.10]
Impaired level of function^e^	Present	44 (93.6)	3 (6.4)	1.24*	14 (56.0)	11 (44.0)	2.64**
	Not/no info	40 (75.5)	13 (24.5)	[1.04–1.49]	14 (21.2)	52 (78.8)	[1.34–5.22]

^a^This refers to the conclusion of legal insanity at the time of the offense committed (in Norwegian legislation “Psychotic” in the legal sense). ^b^RR = Risk ratio. ^c^All other diagnoses from F2 and F3-chapters, including SIPD, see Table 1. ^d^This category includes symptoms recorded as absent, and symptoms not mentioned. ^e^These are general terms used by the forensic experts in their description of a defendant’s mental state

^*^p < 0.05, **p < 0.01, ***p < 0.001

In Tables 3 and 4, symptoms that were described as not present and symptoms that were not described are merged into one number. Table 5 and 6 show symptoms that were recorded as absent and symptoms that were not recorded in addition to the symptoms that were recorded present.

**Table 5:** Symptoms recorded at time of examination with categories present, absent and not mentioned, and active psychotic state, *n* = 191

		Time of examination			
		Schizophrenia		Other psychotic disorders^b^	
**Symptoms**		Active psychotic state^a^		Active psychotic state^a^	
		Yes	No	Yes	No
**P1 Delusions**	Present	60 (88.2)	8 (11.8)	19 (61.3)	12 (38.7)
	Absent	4 (21.1)	15 (78.9)	2 (3.8)	51 (96.2)
	Not mentioned	10 (76.9)	3 (23.1)	0 (0)	7 (100)
**P2 Conceptual disorganization**	Present	49 (87.5)	7 (12.5)	9 (64.3)	5 (35.7)
	Absent	12 (48.0)	13 (52.0)	8 (13.8)	50 (86.2)
	Not mentioned	13 (68.4)	6 (31.6)	4 (21.1)	15 (78.9)
**P3 Hallucina-tory behavior**	Present	47 (90.4)	5 (9.6)	8 (80.0)	2 (20.0)
	Absent	18 (51.4)	17 (48.6)	12 (16.2)	62 (83.8)
	Not mentioned	9 (69.2)	4 (30.8)	1 (14.3)	6 (85.7)
**Psychotic symptoms**^**c**^	Present	55 (88.7)	7 (11.3)	14 (66.7)	7 (33.3)
	Absent	1 (6.7)	14 (93.3)	0 (0)	44 (100)
	Not mentioned	18 (78.3)	5 (21.7)	7 (26.9)	19 (73.1)
**Impaired functioning**^**c**^	Present	44 (84.6)	8 (15.4)	7 (29.2)	17 (70.8)
	Absent	0 (0)	1 (100)	0 (0)	5 (100)
	Not mentioned	30 (63.8)	17 (36.2)	14 (22.6)	48 (77.4)

^a^This refers to the conclusion of whether the defendant had an active psychotic state of illness (equivalent to legal insanity) at the time of the mental examination (MSE). ^b^All other diagnoses from F2 and F3-chapters, including SIPD, see Table 1. ^c^These are general terms used by the forensic experts in their description of the defendants’ mental state

**Table 6:** Symptoms recorded at time of offense with categories present, absent and not mentioned, and legal insanity, *n* = 191

		Time of offense			
		Schizophrenia		Other psychotic disorders^b^	
**Symptoms**		Legal insanity^a^		Legal insanity^a^	
		Yes	No	Yes	No
**P1 Delusions**	Present	33 (89.2)	4 (10.8)	23 (60.5)	15 (39.5)
	Absent	0	0	0 (0)	4 (100)
	Not mentioned	51 (81.0)	12 (19.0)	5 (10.2)	44 (89.2)
**P2 Conceptual disorganization**	Present	15 (88.2)	2 (11.8)	6 (60.0)	4 (40.0)
	Absent	1 (100)	0 (0)	1 (20.0)	4 (80.0)
	Not mentioned	68 (82.9)	14 (17.1)	21 (27.6)	55 (72.4)
**P3 Hallucina-tory behavior**	Present	26 (92.9)	2 (7.1)	8 (53.3)	7 (46.7)
	Absent	0	0	1 (12.5)	7 (87.5)
	Not mentioned	58 (80.6)	14 (19.4)	19 (27.9)	49 (72.1)
**Psychotic symptoms**^**c**^	Present	61 (92.4)	5 (7.6)	15 (48.4)	16 (21.6)
	Absent	1 (11.0)	8 (89.0)	0 (0)	24 (100)
	Not mentioned	22 (88.0)	3 (12.0)	13 (36.0)	23 (64.0)
**Impaired functioning**^**c**^	Present	44 (93.6)	3 (6.4)	14 (56.0)	11 (44.0)
	Absent	0	0	1 (50.0)	1 (50.0)
	Not mentioned	40 (75.5)	13 (24.5)	13 (20.0)	51 (80.0)

^a^This refers to the conclusion of legal insanity at the time of the offense committed (in Norwegian legislation “Psychotic” in the legal sense). ^b^All other diagnoses from F2 and F3-chapters, including SIPD, see Table 1. ^c^These are general terms used by the forensic experts in their description of a defendant’s mental state

### Interrater reliability

We calculated interrater reliability measures for the diagnostic and forensic conclusions and found a Gwet’s AC_1_ value above 0.90, which is considered almost perfect or very good agreement [41, 46]. All symptom variables from the MSE and the PANSS items from the MSO had Gwet’s values above 0.93, which is almost perfect, or very good agreement. Impaired level of function had a Gwet’s value of 0.84 in the MSO, which is still almost perfect, while psychotic symptoms in the MSO had a value of 0.76, which is substantial or good agreement. When analyzing each study year individually (10 reports per year), we found slightly lower agreement for the year 2009. Percent agreement was above 84% for all symptom variables, with most scores above 96%.

## Discussion

### Main findings

In our sample of 500 reports of forensic evaluations from cases of severe violent crime, schizophrenia was the diagnosis most often given when experts gave an opinion of legal insanity. This is in line with other studies, in which the proportions of insanity acquittals associated with a schizophrenia diagnosis were between 49% and 96% [19, 24, 50–57]. Thus, our study supports the link between schizophrenia and legal insanity. Significantly fewer defendants with other psychotic disorders and none with substance induced psychotic disorders were considered legally insane, which underlines the importance of performing a good differential diagnostic evaluation [3]. In Norway, self-induced psychosis by the use of drugs will rarely give legal insanity.

Forensic experts did not systematically describe symptoms of psychosis, be it their presence or absence, in their assessments of defendants’ mental state. They described more such symptoms in their assessments of MSE and considerably fewer in their assessments of MSO, which we find alarming. The incomplete symptom descriptions in experts’ assessments of MSO fuel concerns about the validity of the diagnostic process, and thus the formation of the forensic opinion. Indeed, in the evaluation of legal insanity, symptom presentation and severity are more important than the diagnosis [32]. A diagnostic category is diverse and comprise conditions that are very different in their severity. In addition, diagnostic accuracy and interrater reliability of diagnostic considerations can be quite low [58]. The actual symptoms that affected the defendant at the time of the crime, and how these symptoms affected his or her behavior are more important for the legal consideration than the diagnosis. Even in Norway, where the law does not demand to establish a connection between the symptoms of the illness and the actual criminal act, the experts are asked to show that symptoms of a mental illness affected the defendant to such a degree that his or her ability to have a realistic understanding of their relationship with their surroundings was significantly impaired. That opinions are formed without referring to sufficient data as explanations for the opinions, are one of the frequent errors in forensic report writing [30].

Our results support the concern that experts do not separate the time points of examination and offense in their evaluations, but instead use information from MSE to justify their conclusions about legal insanity at the time of the offense [18, 35]. This is also reported as a common inadequacy of forensic psychiatric investigations [59].

Delusions are central symptoms of the psychotic conditions and diagnoses [42, 60]. The term “delusion” entered the field of criminal responsibility when the lawyer Thomas Erskine used it to justify an insanity verdict in the trial of James Hadfield in England in 1800^4^ [61, 62]. Erskine described delusions as “the true character” and “inseparable companion” of real insanity [4, 61]. Several studies have shown that this symptom is most closely associated with legal insanity and violence, together with hallucinations [16, 18, 21, 23–25, 57, 63, 64]. In the reports we investigated, we found that experts only mentioned the presence or absence of delusions and hallucinations in their assessment of MSO in about one-quarter of the reports. This is in line with the findings from our pilot study [45] and is cause for concern. In the study by Spencer and coworkers, almost 90% of the reports reported delusions as present at the time of the crime [23]. This is considerably more than in our study, where we found that 50% (56 out of 112)^5^ of the reports concluding with a psychosis diagnosis and with legal insanity, mentioned delusions. However, Donohue and coworkers [21] found that 64% of reports where insanity was linked to reduced ability to conform to the law referred to delusions, while 40% of reports that linked insanity to lack of volitional control referred to delusions. This last study, with lower proportions of reports referring to delusions or other symptoms related to psychosis, suggests that our findings may be comparable to findings in reports from other countries. The studies by Spencer et al., and Donohue et al. referred to earlier, found significantly more symptoms mentioned in assessments of MSO than we did. However, the low number of studies on this topic limits comparability between samples.

Experts used the collective term “psychotic symptoms” most often to describe defendants’ MSO. This term was associated with a higher probability of legal insanity both in defendants with schizophrenia and with other psychotic disorders. The experts did not define this collective term in the reports. The construct probably included several of the PANSS items we investigated (delusions, conceptual disorganization, and hallucinations), but it is impossible for the reader of the report to know which. We find it to be of great concern that experts in Norway used this term more than the specific symptoms to describe the defendants symptomatology, as the term leaves the court with little specific information to guide their judgment. Not all symptoms of psychosis are equally important when evaluating the effects of symptoms on MSO, and we advise experts to specify the symptoms they either find to be present or not to be present, and not use a general, unspecified term.

Our most surprising and concerning finding was the difference in the mention of symptoms by diagnosis. Defendants with schizophrenia had the same probability to be considered legally insane if delusions were recorded as present as if delusions were recorded as absent or not recorded at all. Defendants diagnosed with any other psychotic disorders, on the other hand, were declared legally insane 2–6 times more often when delusions and hallucinations were recorded as present at the time of the offense. This indicates that the experts gave a more thorough symptom description when they arrived at the opinion that a defendant diagnosed with other psychotic disorders was legally insane than when they considered a defendant diagnosed with schizophrenia to be legally insane. We find this alarming in terms of the legal security of defendants. Ethical principles of fidelity requires that forensic experts clearly describe the data on which their conclusions were formed. Omitting relevant data is contrary to the integrity principle [65]. Thus, the lack of descriptions of important data for the insanity opinion found in our sample of reports might be a violation of these ethical principles.

In most societies it is seen as a fundamental human and legal right for persons with mental deviances to be granted exemption from criminal responsibility [4]. Nevertheless, to be assessed as a person who cannot take responsibility for one's own actions may be experienced as degrading and stigmatizing for the person in question. Much of the stigma associated with serious mental illness revolves around perceptions of dangerousness [66]. Some argue that to prevent violent acts among persons with schizophrenia will reduce stigma, and lack of responsibility might contribute to this [67]. However, although offenders with schizophrenia are more often considered legally insane, experts must still take into account the narrow definition of legal insanity and use the clinical condition to determine the question of sanity at the time of the offense, not rely solely on a clinical diagnosis [4, 66, 67]. Stigma can also be inherent in societal structures [68]. Each person deserves individual assessment and treatment by the forensic experts, both to preserve their legal and human rights and to adhere to the ethical principles in forensic evaluations [65].

In Norway, the law only requires an assessment of the presence of a severe mental illness at the time of an offense; no connection between that illness and the act must be proven. This is often referred to as the medical or biological principle [10, 41, 69, 70]. Some have expressed concerns that the medical principle in Norwegian legislation, with its emphasis on psychosis, might lead to an even stronger connection between diagnosis and insanity than in legislations with other principles of insanity. Our results give some support to this concern. This might possibly lead to increased stigma of persons with psychotic disorders [24, 71]. Other sources suggest that this is a common challenge faced by forensic experts, regardless of legal principle [1, 3]. A Swedish study of forensic experts’ work methods showed that experts tended to assess many of the aspects of responsibility based on the diagnostic category alone [72]. Several papers and textbooks emphasize that to equate a diagnostic category with insanity is a common error in insanity evaluations [1, 3, 16, 32]. Other studies have also found incomplete clinical descriptions in different kinds of forensic psychiatric evaluations [14, 40].

One possible explanation for our findings may be that forensic experts are influenced by the stigmatized perception that persons with schizophrenia are generally not responsible for their actions [71]. When this diagnosis is presented early in the evaluation process, experts may reach a forensic conclusion of legal insanity without gathering thorough evidence to demonstrate that the severity of the condition is consistent with insanity. This may be a result of judgment biases, e.g., availability bias, anchoring bias, or confirmation bias [31, 35]. Another explanation could be that experts see people with schizophrenia as unfit to be in prison, and that the experts use the possible penal reaction as the most important justification for the insanity suggestion.

### Strengths

This study is one of few that explores the description of psychotic symptoms in reports of forensic evaluations of criminal responsibility regarding legal insanity. To our knowledge, this is the first study with a larger sample size to explore the associations between the symptoms mentioned and the expert opinion of legal insanity in two diagnostic groups. There has been a call for studies that not only report diagnostic categories in forensic reports and their relation to insanity, but also reports the clinical presentations of a defendant, with special attention to the presence of delusions and hallucinations [51]. We believe our study fulfills this expressed need. A sample of 500 reports covering a 10-year period strengthened the internal validity of the design. Moreover, we found no studies that scrutinized symptom descriptions in both sane and insane offenders, and no research that compared symptom descriptions between different diagnostic groups. Our use of a generalized linear mixed model may be seen as a strength as these models are recommended in psychiatric and psychological research when data are not normally distributed [73].

### Limitations

The PANSS was developed as a research tool to monitor changes in symptoms of psychosis during treatment. We modified the use of this instrument to condense and extract written information on targeted symptoms. Although interrater reliability was good for this use of the PANSS, further use would require subsequent reliability testing. Another limitation is that the category “other psychotic disorders” is very diverse, including schizotypal disorders, paranoid psychosis, acute psychosis, affective psychoses, and SIPD.

The chosen selection criterion severity of the offense was another limitation. As our sample was selected based on indictment, some experts wrote many reports while others wrote few. Standard regression models assume independent observations. Since the experts wrote more than one report, the reports were clustered within expert teams, and the assumption of independence was violated. To compare for this limitation, an inter-cluster correlation was introduced for the pair of experts and the risk ratios were analyzed by general linear mixed models. The estimations of risk ratios were based on low cell counts in some cases, which might give non-robust estimates. Even moderate changes in cell counts might lead to considerable differences in the risk ratios.Our study was not designed to validate symptom descriptions, as we did not study the actual evaluation process. Although a validation study of the evaluation process might be of interest, we focused on the reports because they are used as evidence in courts. The interrater agreement for the symptoms in the reports was very high. Therefore, the observed differences in symptom assessment between persons with schizophrenia and those with other psychosis may be considered both reliable and valid.

Symptom descriptions might also be associated with other features, such as the length of the reports, collateral information collected, whether the defendant cooperated with the experts, and the time interval between the crime and the first interviews. We did not include these features in our analyses, which may be considered a limitation of our study.

### Clinical implications

Our results are of such concern that we believe they should provoke improvements in the clinical practice and in the writing procedure and routine of forensic experts; at least in Norway. It is important to make sure that experts report not only on diagnostic conclusions, but also on the symptoms relevant to the question of insanity. As other studies have indicated that this problem might not be limited to the Norwegian legal system, we believe our findings should inspire more research and quality improvement regarding the description and reporting of psychotic symptoms in forensic reports in other countries as well.

When reporting symptoms that was present in MSO, at the time of the crime, the experts will have to search for information in other sources than their own examinations of the defendant. The defendant might provide important information about his or her condition, but they might also have a self-interest in exaggerating or hiding this information [3, 7]. We believe the experts should be more thorough in finding evidence of symptoms present in the collateral information they collect, as this is a core task in forensic evaluations of criminal responsibility [1, 3, 30].

### Future research

We think it would be interesting to see similar studies performed in countries where a connection between the symptoms present and the offense committed must be proven for a defendant to be considered legally insane. This could improve understanding of how a legal principle affects clinical examinations and descriptions. The legal principle for legal insanity in Norway changed after we selected the reports for this study [10, 12]. To replicate this study using reports written after this change may provide important insight into whether changes in the rule of law affect the clinical practice of forensic experts.

As many forensic experts in Norway also work in clinical settings, our results raise some concerns regarding how mental health personnel in general report on symptom presentations when they consider different diagnoses for their patients. We believe our findings may be relevant for the general mental health practice as well, and similar studies as ours might be conducted with written medical records from clinical mental health care settings.

Future research should analyze how the descriptive features of the reports, such as the report length, are associated with symptom descriptions. When performing research with correlational analyses, the experts that author the reports could be a selection criterion, for a better understanding of actual differences between the experts’ methods. Moreover, it would be interesting to know more about which information the judges refer to in their verdicts when presented with forensic witnesses’ reports and oral statements in court. Investigations have shown that courts usually follow the experts’ conclusions in their verdicts [10]. The experts’ reporting of symptom descriptions from collateral sources could be studied if the researchers recorded which symptoms they found in these sources and compared them with the experts’ descriptions in the reports.

## Conclusion

We showed that Norwegian forensic experts did not systematically mention either the presence or absence of symptoms important for psychotic disorders in their reports regarding criminal responsibility. Symptoms were mentioned less often in defendants diagnosed with schizophrenia than in defendants diagnosed with other psychotic disorders. This raises concerns regarding the validity and reliability of forensic evaluations of legal insanity, and for the legal security of offenders with mental illness. When experts do not present sufficient data, judges may depend too much on the diagnostic categories and the experts’ conclusions. It is concerning that offenders with schizophrenia are considered insane without an individual assessment of their symptoms or functioning at the time of the offense [74]. The risk of stigma associated with publicity in cases where people with schizophrenia commit violent crimes adds to the importance that the diagnostic category in itself should not lead to an opinion of legal insanity.

## Data Availability

The datasets generated and analyzed during the current study are not publicly available due to the Data protection policy at Oslo University Hospital. They are stored in a secured research server and are available from the corresponding author on reasonable request.

## Footnotes

1 The rule of law changed in 2020, and the legal construct is now “strongly deviant state of mind”.

2 The rule of law and practice of forensic experts changed in 2020, and experts are no longer supposed to give an opinion of the question of responsibility, but only describe the mental condition at the time of the crime.

3 In some countries, the mental state at the time of the evaluation is almost as important as the time of the crime, as the penal reactions to the offense is the focus of the evaluation more than the principle of freedom of liability.

4 This trial was one of the first to establish insanity as a special verdict, and the first to use delusions as a justification.

5 Numbers from Table 4.
